# Predictors of COVID-19 vaccine hesitancy among Egyptian healthcare workers: a cross-sectional study

**DOI:** 10.1186/s12879-021-06392-1

**Published:** 2021-08-05

**Authors:** Rehab H. El-Sokkary, Omnia S. El Seifi, Hebatallah M. Hassan, Eman M. Mortada, Maiada K. Hashem, Mohamed Rabie Mohamed Ali Gadelrab, Rehab M. Elsaid Tash

**Affiliations:** 1 Medical Microbiology and Immunology Department, Faculty of Medicine, Zagazig University, Ash Sharqiyah, Egypt; 2 Community, Occupational and Environmental Medicine Department, Faculty of Medicine, Zagazig University, Ash Sharqiyah, Egypt; 3 Department of Family and Community Medicine, Faculty of Medicine, University of Tabuk, Tabuk, Kingdom of Saudi Arabia; 4 Medical Microbiology and Immunology Department, Faculty of Medicine, Assiut University, Assiut, Egypt; 5 Health Sciences Department, Health Sciences & Rehabilitation College, Princess Nourah Bint Abdulrahman University, Riyadh, Saudi Arabia; 6 Chest department, faculty of Medicine, Assiut University, Assiut, Egypt; 7 Anaesthesia and Intensive Care Medicine at Agouza Police Hospital, Giza, Egypt

**Keywords:** COVID-19 pandemic; COVID-19 vaccine, Vaccine hesitancy, Second wave, Egypt

## Abstract

**Background:**

Coronavirus disease 2019 (COVID-19) vaccination has raised concerns about vaccine hesitancy in general and COVID-19 vaccine hesitancy in particular. Understanding the factors driving the uncertainty regarding vaccination against COVID-19 is crucial.

**Methods:**

This cross-sectional study was designed to identify the perceptions and attitudes of healthcare workers (HCWs) towards COVID-19 vaccines and determine the predictive factors that affect their willingness to receive the COVID-19 vaccine. An online survey was distributed among HCWs to collect data assessing demographic and general characteristics of the participants and vaccine-related characteristics, including source of information about the vaccine. In addition to items assessing the perception of COVID-19, there were items on COVID-19 vaccines and attitude towards vaccination in general and towards COVID-19 vaccines in particular.

**Results:**

The participants were classified according to their willingness to take the COVID-19 vaccine as follows: hesitant (41.9%), refusing (32.1%), and willing (26%). Statistically significant differences were observed among the three groups for the perception of COVID-19 vaccines, attitude towards vaccination in general, and COVID-19 vaccines in particular (*p* < 0.01).

**Conclusions:**

Although the participants adequately perceived COVID-19 severity, prevention, and COVID-19 vaccine safety, they were widely hesitant or refused to be vaccinated. A multidimensional approach is required to increase the vaccine acceptability rate. Higher income and increased years of work experience are positive predictors of willingness to receive a vaccine. Thus, further studies addressing the scope of COVID-19 vaccine hesitancy are warranted as an initial step to build trust in COVID-19 vaccination efforts with continuous monitoring of attitudes and practices of HCWs towards COVID-19 vaccines in the future.

**Supplementary Information:**

The online version contains supplementary material available at 10.1186/s12879-021-06392-1.

## Background

One of the greatest public health achievements in the twentieth century is vaccination [1]. During the coronavirus disease 2019 (COVID-19) pandemic, the role of healthcare workers (HCWs) in pandemic control became more prominent [2]. Low vaccination acceptance rates among HCWs can decrease the vaccination compliance of individuals who coincidentally engage with vaccine-hesitant HCWs at a professional or personal level. This finding is of concern because HCWs are the most reliable social resource to encourage vaccination among the general population [3]. They are in the best position to understand and respond to worries and concerns of hesitant patients and explain to them the benefits of vaccination. However, numerous recent studies have shown that HCWs themselves, including those who provide patient vaccination, can be vaccine hesitant, not only towards vaccination for themselves, but also towards vaccination of their children or their patients [2].

As of February 18, 2021, at least seven COVID-19 vaccines have been developed [4]. COVID-19 vaccination raised many concerns about hesitancy in vaccines in general and COVID-19 vaccines in particular. The delay in acceptance or the refusal of vaccines, despite the availability of vaccination services, is referred to as ‘vaccine hesitancy’, which is complex and context-specific, varying across time, place, and specific COVID-19 vaccines. Many aspects influenced hesitancy, including confidence, the level of trust in the vaccine or provider, complacency, the lack of perception of the need for a vaccine or value of the vaccine, convenience, and access issues [5]. Understanding the factors driving uncertainty regarding vaccination against COVID-19 is crucial.

As of March 3, 2021, a total of 1315 vaccine doses have been administered in Egypt [6], Few studies have measured COVID-19 vaccine acceptance among potential HCWs in Egypt. Two studies enrolled medical students as participants, both registered vaccine acceptance lower than that in Western countries, but higher than that in African countries [7, 8]. There are few published reports to date regarding HCWs’ acceptance rate of COVID-19 vaccines. Thus, we conducted this study to identify the perceptions and attitudes of HCWs towards COVID-19 vaccines and determine predictive factors for willingness to receive the COVID-19 vaccine.

## Methods

A cross-sectional study was conducted using surveys disseminated online in Egypt, a middle-income country in northeast Africa, during the second wave of COVID-19 in January 2021. At that time, vaccine availability in Egypt was restricted, vaccination campaigns had not yet been initiated, and only HCWs were eligible for vaccination. The survey was aimed at Egyptian HCWs from different specialties and healthcare sectors. The study was conducted according to the international guidelines of Strengthening the Reporting of Observational Studies in Epidemiology (STROBE) [9].

Using Open Epi, the sample size was calculated to be 308, as the rate of COVID-19 vaccine acceptance among HCWs was 27.7% in a similar study at a confidence interval (CI) of 95%. The power of the test was 80%, and the design effect was 1 [10]. Data were collected anonymously via the online survey that was designed after a review of the literature [5, 11–13]. The questionnaire was revised and then pilot tested on 30 HCWs to determine the acceptability and clarity of the questions and confirm its face validity and it was modified accordingly. The responses obtained in the pilot study were not included in final analysis. Internal consistency was measured; Cronbach’s coefficient was 0.83.

The questionnaire was prepared, distributed, and collected using Google Forms. A web-based URL was created through which respondents accessed the survey and submitted their responses. For the distribution, a convenient sampling method was adopted. The URL was posted via the network of the research team and the HCWs’ professional groups on WhatsApp, Facebook, and Facebook Messenger from January 25 to 31, 2021. The following statement was included in the heading of the questionnaire:” This questionnaire is administered for research purposes only. Confidentiality of data is guaranteed. By submitting your answer, you give the researchers your consent to participate in this research work.” The questionnaire consisted of four parts: (1) demographic and general characteristics of the participants including their current job status (during the COVID-19 pandemic); (2) vaccine-related characteristics, including source of information about the vaccine; (3) perception about COVID-19 severity (six items) and COVID-19 vaccine safety (eight items); and (4) attitude towards vaccines and vaccination in general (eight items) and COVID-19 vaccines in particular (eight items).

To assess HCWs’ perception towards COVID-19, answers were scored as follows: agree, 2 points; neutral, 1 point; and disagree, 0. The total score ranged from 0 to 12. Perception regarding the COVID vaccine was assessed using eight questions, with a total score ranging from 0 to 16. A perception level of ≥60% was considered as adequate perception [14]. To assess HCWs’ attitudes towards vaccines and vaccination in general and their attitude towards COVID-19 vaccines in particular, eight questions were used to assess each area, using the same principle as that for perception. The total score of the responses ranged from 0 to 16 for each area. A level of attitude ≥60% was considered a positive attitude [14].

### Outcome measure

To measure the vaccination intention, we asked the participants to state their intention to undergo COVID-19 vaccination on a three-point scale: “agree, neutral, or disagree”.

### Statistical analysis

Data were entered, coded, and analysed using SPSS 23 (IBM Corp., Armonk, NY, USA). Descriptive statistics were obtained by calculating the mean and standard deviation for quantitative data and the number and percentage for qualitative and discrete data. Chi-square was conducted to find an association between the different variables between the groups. The Kruskal-Wallis test was used to assess the differences between the mean perception and attitudes of the three groups of participants. Spearman correlation analysis was performed to assess the correlation between the level of attitude towards COVID-19 vaccines and the different levels of perception and attitude. Multinomial regression was conducted to determine the predictive factors for the willingness to administer the COVID-19 vaccine. Differences were considered statistically significant at *p* ≤ 0.05.

## Results

**Fig. 1:**
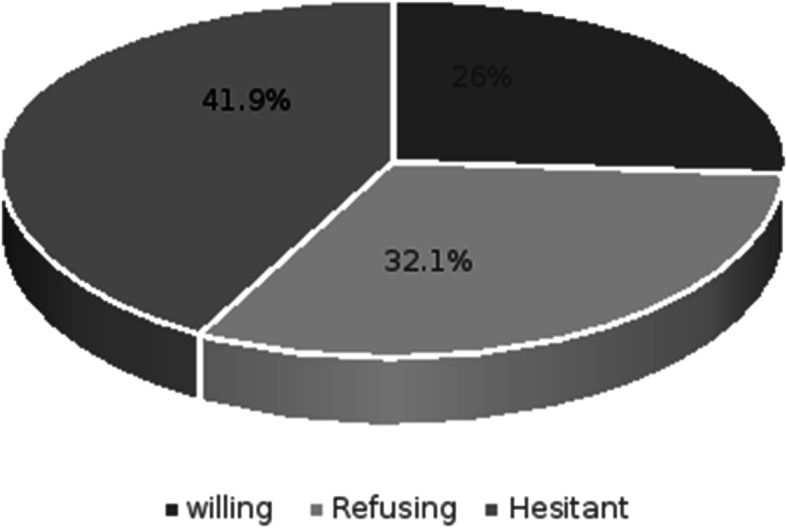
Intention of the participants to get the COVID-19 vaccine. Shows the distribution of HCWs’ status towards COVID-19 vaccine into hesitant, refusing and accepting

A total of 308 responses were analysed. The mean age was 37.6 ± 10.1 years, most participants were female (77.6%), married (80.8%), had an income < 2000 LE/month (60.1%), were physicians (47.4%), had direct patient contact (51.9%), had more than 10 years of experience (53.2%), and had no comorbidities (74.7%). Furthermore, some participants had a medical doctorate degree (29.2%) and worked in university hospitals (33.1%). The participants were classified according to their willingness to take the COVID-19 vaccine as follows: hesitant (41.9%), refusing (32.1%), and willing (26%), as illustrated in Fig. 1.

Table 1 demonstrates the sociodemographic and general characteristics of the three study groups. Significant differences were found among them with regard to age (*p* < .001), sex (*p* = .001), level of education, income level (*p* < .001), and years of work experience (*p* = .002).

**Table 1:** Sociodemographic and general characteristics of the participants’ study groups

Items	Category	Willing N. 80 (26.0%)	Hesitant N.129 (41.9%)	Refusing N. 99 (32.1%)	***P*** value
**Age** ^**H**^	(Mean ± SD)	42.5 ± 12.2	35.1 ± 7.9	37.0 ± 9.5	<.001*
**Sex**	Male	29 (36.2)	26 (20.2)	14 (14.1)	.001*
	Female	51 (63.8)	103 (79.8)	85 (85.9)	
**Marital status**	Single	8 (10.0)	22(17.1)	13 (13.1)	.07
	Married	72 (90.0)	98(76.0)	79 (79.8)	
	Divorced/widow	0 (0.0)	9 (7.0)	7 (7.1)	
**Level of Education:**	Bachelor’s degree	6 (7.5)	37 (28.7)	21 (21.2)	<.001*
	Diploma level	10 (12.5)	27 (20.9)	19 (19.2)	
	Master’s degree	17 (21.2)	36 (27.9)	31 (31.3)	
	Medical doctorate	41 (51.2)	25 (19.4)	24 (24.2)	
	Egyptian Fellowship	4 (5.0)	4 (3.1)	2 (2.0)	
	Others	2 (2.5)	0 (0.0)	2 (2.0)	
**Income** /month	< 2000 LE.	2 (2.5)	7 (5.4)	27 (27.3)	<.001*
	2000–5000 LE	32 (40.0)	98 (75.0)	62 (62.6)	
	> 5000 LE/month	46 (57.5)	31 (24.0)	32 (32.3)	
**Profession**	Physician	52 (65.0)	69 (53.4)	64 (64.7)	.12
	Dentist	5 (6.2)	13 (10.1)	4 (4.0)	
	Pharmacist	13 (16.3)	38 (29.5)	23 (23.2)	
	Others	10 (12.5)	9 (7.0)	8 (8.1)	
**Current Job status during COVID-19**	I have a direct patient contact.	40 (50.0)	75 (58.1)	72 (45.5)	.34
	I do not have a direct patient contact.	38 (47.5)	50 (38.8)	52 (52.5)	
	I am currently not practicing medicine	2 (2.5)	4 (3.1)	2 (2.0)	
**Year of working experience**	<five years	3 (3.8)	25 (19.4)	16 (16.2)	.002*
	5–10 years	24 (30.0)	49 (38.0)	27 (27.3)	
	> 10 years	53 (66.3)	55 (42.6)	56 (56.6)	
**Place of work**	Private Hospital/clinic	3 (3.7)	7 (5.4)	6 (6.1)	.43
	MOHP Hospital	12 (15.0)	28 (21.7)	17 (17.2)	
	University Hospitals	32 (40.0)	36 (27.9)	33 (33.3)	
	Teaching Hospitals	17 (21.3)	23 (17.8)	11 (11.1)	
	Health insurance Hospitals	2 (2.5)	8 (6.2)	7 (7.1)	
	Others	14 (17.5)	27 (21.0)	25 (25.2)	
**Comorbidities**	Hypertension	7 (8.8)	5 (3.9)	7 (7.1)	.06
	Diabetes mellitus	4 (5.0)	3 (2.3)	5 (5.1)	
	Heart disease	5 (6.3)	4 (3.1)	0 (0.0)	
	Lung diseases	1 (1.3)	3 (2.3)	1 (1.0)	
	Renal diseases	0 (0.0)	2 (1.6)	0 (0.0)	
	Autoimmune diseases	2 (2.5)	1 (0.8)	5 (5.1)	
	Immunosuppressive disorders	0 (0.0)	0 (0.0)	1 (1.0)	
	None	58 (72.5)	104 (80.6)	68 (68.7)	
	Other	3 (3.8)	7 (5.4)	12 (12.1)	

Chi square was computed. * *p* ≤ .05 is significant, H = Kruskal–Wallis test

Table 2 shows vaccine-related characteristics among the participants according to the vaccine willingness categories. A significant difference was reported between the groups regarding the source of information about COVID-19 vaccines, wherein 85% of the participants who were willing to take the COVID-19 vaccine depended on international websites, such as the World Health Organization (WHO) and Centers for Disease Control and Prevention (CDC), as their source of information about COVID-19 vaccines (*p* < .001). Attendance of scientific meetings about vaccine benefits encouraged vaccine administration in most HCWs in the willing group, with a significant difference between the three groups (*p* < .001). Following COVID-19 vaccine news in the media, most HCWs in the willing group (90.1%) were motivated to take the vaccine (*p* < .001).

**Table 2:** Vaccine-related characteristics among the participants according to the vaccine’s willingness categories

Items		Willing ***N*** = 80 No (%)	Hesitant ***N*** = 129 No (%)	Refusing ***N*** = 99 No (%)	***P*** value
**Source of information about COVID-19 vaccine**	**-Social media:**				
	Yes	27 (33.8)	61 (47.3)	41 (41.4)	.15
	No	53 (66.3)	68 (52.7)	58 (58.6)	
	**-TV:**				
	Yes	12 (15.0)	28 (21.7)	24 (24.2)	.30
	No	68 (85.0)	101 (78.3)	75 (75.8)	
	**-Official website of MOHP:**				.48
	Yes	34 (42.5)	63 (48.8)	41 (41.4)	
	No	46 (57.5)	66 (51.2)	58 (58.6)	
	-**International organizations** **websites (WHO, CDC):**				<.001*
	Yes	68 (85.0)	74 (57.4)	63 (63.6)	
	No	12 (15.0)	55 (42.6)	36 (36.4)	
**Attendance of courses regarding COVID-19 vaccine**	Yes	42 (52.5)	54 (41.9)	36 (36.4)	.09
	No	38 (47.5)	75 (58.1)	63 (63.6)	
**Attendance of meeting discussion on vaccine benefits**	-Recommending the vaccine	32 (88.9)	12 (48.0)	7 (33.3)	<.001*
	-Discouraging the use of the vaccine	4 (11.1)	13 (52.0)	14 (66.7)	
**Following COVID-19 vaccine news in media**	-Encourage you to take the vaccine	64 (90.1)	49 (50.0)	10 (14.5)	<.001*
	-Discourage the use of the vaccine	7 (9.9)	49 (50.0)	59 (85.5)	
**Hearing that all available COVID-19 vaccines are under the umbrella of (EUA)**	-Encourage you to take the vaccine	38 (47.5)	22 (17.1)	12 (12.1)	<.001*
	-Discouraging the use of the vaccine	24 (30.0)	38 (29.5)	39 (39.4)	
	-I did not hear about EUA	18 (22.5)	69 (53.4)	48 (48.5)	

* *p* ≤ .05 is significant, *MOHP* Ministry of Health and Population, *EUA* emergency use authorization

Knowing that all of the available vaccines for COVID-19 are under the umbrella of emergency use authorisation (EUA) encouraged 47.5% of the participants in the willing group to take the vaccine. Alternatively, 53.4 and 48.5% of the participants in the hesitant and refusing groups, respectively, had not heard about EUA, versus 22.5% in the willing group (*p* < .001).

Significant differences were observed among the three groups regarding the perception of COVID-19 vaccine safety, attitude towards vaccination, and COVID-19 vaccination (*p* < 0.01) (Table 3).

**Table 3:** Mean perception and Mean attitude of the participants to COVID 19 pandemic and vaccines

Items	Willing Mean ± SD	Hesitant Mean ± SD	Refusing Mean ± SD	***P*** value
**Total Perception to COVID-19 infection**	**3.60 ± 1.7**	**4.16 ± 2.2**	**4.17 ± 2.4**	**0.302**
**Total Perception to COVID-19 vaccine**	**6.40 ± 2.6**	**7.82 ± 2.2**	**8.77 ± 2.5**	**0.000**
**Total Attitude to vaccines and vaccination in general**	**7.78 ± 2.1**	**7.01 ± 2.2**	**6.17 ± 2.9**	**0.000**
**Total Attitude to COVID-19 vaccine**	**7.66 ± 1.6**	**6.58 ± 2.4**	**4.9 ± 3.1**	**0.000**

Kruskal-Wallis was computed. *p* ≤ 0.05 is significant

**Fig. 2:**
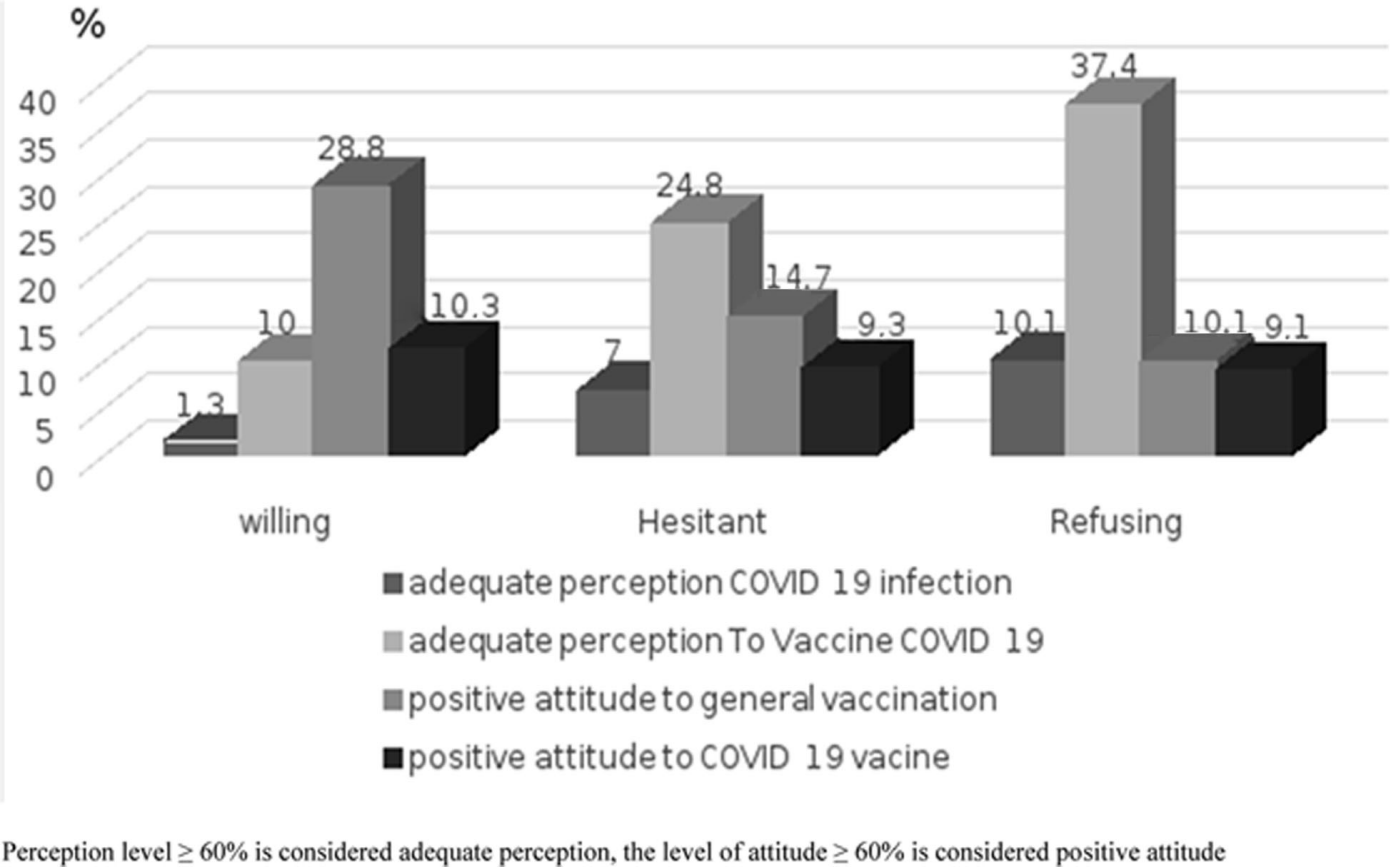
Distribution of an adequate level of perception to COVID-19 infection and vaccination, a positive attitude toward general vaccination and to COVID-19 vaccine among different participants’ categories

Adequate levels of perception for the severity of COVID-19 and for COVID-19 vaccine safety were higher in the refusing group (10.1 and 37.4%, respectively), while they were the lowest in the willing group (1.3 and 10% for the willing and hesitant groups, respectively). The adequate levels of both attitudes towards vaccination in general and COVID-19 vaccination in particular were higher in the willing and hesitant groups (28.8 and 11.3%, respectively) and the lowest in the refusing group (10.1 and 9.1%, respectively) (Fig. 2). Responses to individual items are presented in Supplement 1.

The willingness to take the COVID-19 vaccine was significantly positively correlated with the HCWs’ perception of the severity of COVID-19 (*r* = 0.316, *p* = 0.004) and COVID-19 vaccine safety (*r* = 0.277, *p* = 0.013). Hesitancy and refusal to take vaccines were significantly positively correlated with attitudes toward vaccines and vaccination in general (*r* = 0.469, *p* < 0.01, and *r* = 0.477, *p* < 0.01, respectively).

The significant predictive factors for the intention to administer the COVID-19 vaccine among the participants were income (odds ratio [OR], 11.96; CI, 1.917–4.628; *p* = 0.008) and years of experience (OR, 11.465; CI, 1.352–7.212; *p* = 0.025) (Table 4).

**Table 4:** Significant Predictors of the intention to get COVID-19 vaccines among the participants

Variables	B	SE	***P*** value	Expected B	95.0% C I for expected B	
					Lower bound	Upper bound
Income	2.48	0.93	0.008*	11.96	1.92	4.63
Years of experiences	2.439	1.091	0.025*	11.47	1.35	7.21

* *P* ≤ 0.05 is significance; B:unstandardized beta” regression coefficient”; β:standardized beta,

## Discussion

In 2019, the WHO identified 10 threats to global health, including vaccine hesitancy and the risk of a pandemic [15]. A challenge for healthcare leaders is to discern the origin of HCWs’ vaccine hesitancy. In this study, we explored the perception and attitude of HCWs towards COVID-19 vaccines and identified the predictive factors that affect their willingness to undergo COVID-19 vaccination. We conducted this evaluation before the mass vaccination for COVID-19 started in Egypt.

Nearly one-third of our participants agreed to be vaccinated. Different rates of readiness to take the vaccine, either high or low, were reported earlier. For example, willingness to get vaccinated ranged from 60 to 90% among physicians in Greece (February 2020) [16], France (March–July 2020) [11], France and French-speaking parts of Belgium and Canada [5], Turkey (September 2020) [12], and KSA (November 2020) [17].

In contrast to the high acceptance rate, an earlier study in Congo (March–April 2020) reported that only 28% of HCWs were willing to take a vaccine when the vaccines would be made available [10]. A previous study conducted among Egyptian HCWs (December 2020) reported an acceptance rate of 46% [8]. Shekhar et al. reported that about one-third (36%) of surveyed US HCWs were willing to receive the COVID-19 vaccine as soon as it became available (October–November 2020) [18]. The effect of time should be considered to rationalise the low acceptance rate among HCWs [19]. COVID-19 vaccine willingness can change substantially with time, experience with actual vaccine administration, and the time-varying morbidity and mortality values of the ongoing pandemic [20]. In this study, we conducted this assessment during the second wave of the pandemic, just before the actual start of vaccine administration, and the CDC/WHO approval of all available vaccines in Egypt.

In the present report, demographic characteristics showed significant differences in age, sex, level of education, income, and work experience. Most respondents were female (70%), which was a contributing factor to the low vaccine acceptance rate. It has been previously observed that women are less accepting of vaccination [20, 21]. Shekhar et al. also noticed the same association where vaccine acceptance increased with increasing age, education, and income level, as well as lower acceptance in females [18]. Gain-framed messages are effective in promoting certain types of prevention [22]. This result could direct healthcare authorities to design different vaccine messages tailored according to the target audience.

Dolan et al. and Meyer et al. noted that the source of information influenced the extent to which recipients incorporate the information into their decision-making process [23, 24]. In this study, international organisation websites were the main source of information for the willing group. However, the source was not found to be the only contributing factor. Trust in the institutions through which information about vaccines is delivered is an essential driver of vaccine acceptance, for not only the general population, but also HCWs [25].

In addition, the impact of the media cannot be underestimated. We noticed that following the vaccine news in the media, there was a significant difference between the willing and hesitant HCWs. This observation underscores the harm of the spread of misinformation across the media, which the WHO named the “infodemic” (i.e. excessive amounts of misinformation and rumours that make it difficult identify reliable sources of information) [26]. At that time in Egypt, we lacked proper messages delivered across media [27].

The fact that all available COVID-19 vaccines are under the umbrella of EUA was expected to be a discouraging factor; however, about 48% of the willing group were aware of this fact and still accepting of the vaccine. Providing HCWs with information explaining this vague term can encourage them to accept vaccination [5].

HCWs have a positive attitude towards vaccines because they have scientific and medical training. Nevertheless, HCWs are not a homogenous group and most are not experts in the field of vaccination [28]. Moreover, immunisation is not an important part of initial training [29]. In fact, the emergence of COVID-19 vaccines revealed a knowledge gap in immunological sciences among physicians, which is another issue to be explored in further studies.

It is not a single factor, but a mixture of several factors that affect the attitude towards the acceptance of vaccination [30]. In this study, although the participants adequately perceived COVID-19 severity and prevention strategies as well as COVID-19 vaccine safety, they were widely hesitant to be vaccinated. Other factors underlying this hesitancy or refusal should be examined.

It is crucial to assess the predictors of vaccine uptake among HCWs, which will help health authorities and policymakers target resources to maximise uptake. In the current study, income and years of experience were found to be significant predictive factors for the willingness to administer COVID-19 vaccines among the participants. The predictors varied for different populations of HCWs who responded to similar surveys in different parts of the world: willingness to receive influenza vaccinations in ordinary years [3, 5] and individuals who categorised themselves as being at high risk for severe COVID-19 [3].

### Limitations of the study

Using a convenient sampling technique limits the generalizability of the study findings and might create selection bias. The study was conducted when COVID-19 vaccines were not yet introduced to HCWs in Egypt, and it is possible that the actual introduction of the vaccine will alter the acceptance rate.

## Conclusion

Although participants adequately perceived COVID-19 severity issues as well as COVID-19 vaccine safety, they were widely hesitant or refused to be vaccinated. Statistically significant differences were reported for the perception of COVID-19 vaccine safety, attitude towards vaccination in general, and attitude towards COVID-19 vaccinations in particular (*p* < 0.01). The significant positive predictive factors for the intention to administer and receive COVID-19 vaccines among the participants were higher income and more years of experience. A multidimensional approach is needed to increase the vaccine acceptance rate. Further research is highly recommended to address the scope of COVID-19 vaccine hesitancy as an initial step for building trust in COVID-19 vaccination efforts. Continuous monitoring of attitudes and practices of HCWs towards COVID-19 vaccines in the period ahead is recommended.

## Supplementary Information

**Additional file 1: Figure S1.** Shows the details of responses (Mean score) to different questionnaire items as regards perception to COVID-19. **Figure S2.** Shows the details of responses to different questionnaire items as regards perception to COVID-19 vaccine. **Figure S3.** Shows the details of responses to different questionnaire items as regards attitude to vaccine and vaccination in general. **Figure S4.** Shows the details of responses to different questionnaire items as regards attitude to COVID-19 vaccines.

## Data Availability

The data sets used and/or analyzed during the current study are available from the corresponding author on reasonable request.
