# Supplementary material for: Predictors of COVID-19 vaccine hesitancy among Egyptian healthcare workers: a cross-sectional study
Source: BMC Infect Dis. 2021 Aug 5;21:762. doi: 10.1186/s12879-021-06392-1 (PMC8341553; doi:10.1186/s12879-021-06392-1)
Supplement: Supplementary file 1 — Additional file 1: Figure S1. Shows the details of responses (Mean score) to different questionnaire items as regards perception to COVID-19. Figure S2. Shows the details of responses to different questionnaire items as regards perception to COVID-19 vaccine. Figure S3. Shows the details of responses to different questionnaire items as regards attitude to vaccine and vaccination in general. Figure S4. Shows the details of responses to different questionnaire items as regards attitude to COVID-19 vaccines. [file 12879_2021_6392_MOESM1_ESM.docx]

Figure S1: Individual questionnaire items (Perception to COVID-19):

**Figure S1 shows the details of responses (Mean score) to different questionnaire items as regards perception to COVID-19**


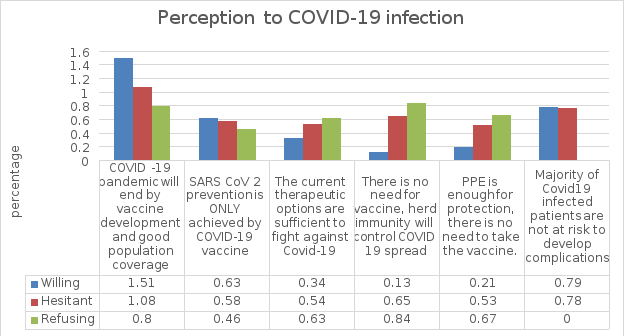


Mean score

Figure S2: Individual questionnaire items (Perception to COVID-19 vaccine):

**Figure S2 shows the details of responses to different questionnaire items as regards perception to COVID-19 vaccine.**


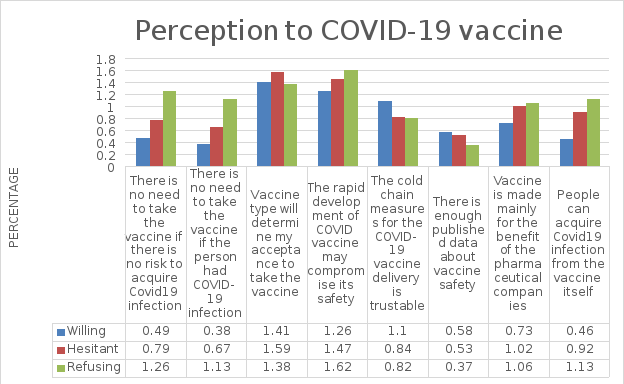


Mean score

Figure S3: Individual questionnaire items (Attitude to vaccine and vaccination):

**Figure S3 shows the details of responses to different questionnaire items as regards attitude to vaccine and vaccination in general**


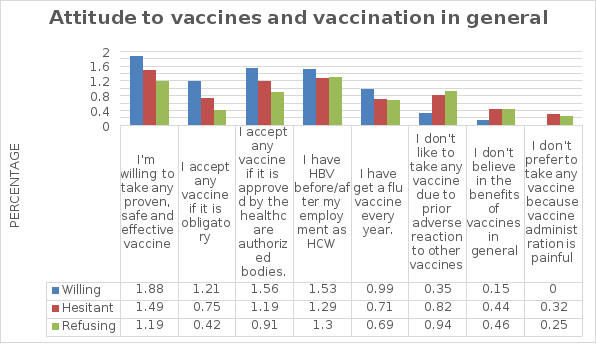


Mean score

Figure S4: Individual questionnaire items (Attitude to COVID-19 vaccines):

**Figure S4 shows the details of responses to different questionnaire items as regards attitude to COVID-19 vaccines.**


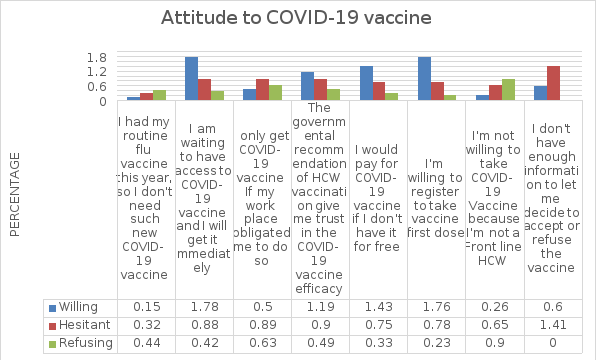


Mean score
